# Skimble-Scamble “Dots” by the Wayside

**Published:** 1876-10

**Authors:** Thin Pate

**Affiliations:** Berea, N. C.


### SOUTHERN MEDICAL RECORD.

VOL. VI. OCTOBER, 1876. No. 10

### Original Communications.

### SKIMBLE-SCAMBLE "DOTS" BY THE WAYSIDE.

BY THIN PATE, M. D., OF BEREA, N. C.

*A Case.*—"Old grannies" have not been laid aside in some neighborhoods, as worn-out tools from the hands of the Devil, as will appear from the following case. By "grannies" is generally, in this country, only meant the ignorant female midwives of the country, but here I also mean those "sage feminines" (?) who can tell you how old Betsy Ann is by *her* thirteenth child's age; can talk "FIRE" out of a burn; will tell you to stew "smart weed" and lard for "scald-head;" give "catnip" tea for "little hives," or can produce labor pains by piling men's hats on a woman's head. *Appropos*, I may remark that these are no exaggerated surmises, for I have listened patiently (?) to the prescriptions, wished secretly for "the fool killer" for the fire witch, (?) and had the pleasure of knocking the talismanic hats to the four corners of a log cabin. But to begin the case: On the night of March —, 1876, I was called in great haste to Mrs. ——— in labor. I was soon on the scene of action—but not to act much—to find the woman on her husband's lap groaning under powerful "bearing down" pains, while an "old grannie" sat between her knees with both hands under her clothing, quietly saying, "the head is against the cross-bones." Knowing the meaning of all this, and that the last stages of labor were being accomplished, I did nothing but took my stand, with one arm akimbo and the other hand resting on the back of a chair, simply a "looker on in Venice." In a few minutes the child was delivered; the placenta followed immediately, and the following directions were minutely given and as minutely followed: "Now stand up, lock your hands over your head, and *count three*; now take a long breath, blow *three times* through your fist; now go to bed." A *fried egg* was then applied to the external parts. She was made to lay on her left side and receive the whole weight of the buxom midwife "to press the cross-bones together." She was then thickly covered, and left to rest in her glory.

"*Oil of Butter*."—In the summer of 1875, while attending a child with chronic diarrhœa, an old lady, the child's grandmother, gave it "oil of butter" with paregoric during my absence, saying it wonld certainly cure it, etc. I immediately stopped it on my return, and thought of it only in disgust until April, 1876. Having a very severe case of entero-collitis, produced by artificial diet. After trying several remedies, apparently with no effect, in looking over my journals, I came across the article by Dr. T. Curtis Smith, in SOUTHERN MEDICAL RECORD (December No. of 1875,) which suggested to me the *rationale* of using "oil of butter," which I accordingly did, ordering it as follows: Place the butter (ordinary butter from cow's milk) in a bowl, and pour BOILING water on it. Allow it to sit until it cools, then skim off all that rises to the top, and give a teaspoonful of this, with ½ to 1 drop of laudanum, or its equivalent of paregoric, every four hours (to child seven months old.) In addition, poultices were applied to the bowels, and warm baths several times daily. On my return the next morning, the child was much improved. The contractions of its hands and feet, which were sufficient for the nails to cut its hands, had relaxed, and the bowels not so tender, and movements somewhat checked. I am inclined to give the butter some credit, because all the other remedies had been employed with no apparent effect. I have since used the "oil of butter" both as a gentle purgative, and with paregoric as a remedy in dysentery and diarrhœa, with good effect, and am told that the late lamented Dr. McKee, of Raleigh, used it years ago in these cases. Of course such experience (so limited, etc.,) cannot establish its merit, yet it has the advantage of being simple, easy to come at, nutritive, and at least deserves notice.

"*Chicken Pepsine.*"—It has been the practice of several physicians of this county to use what we call "chicken pepsine" for several years past. I do not know who introduced it, but I have found it more satisfactory than the generality of the prepared pepsine we get in the shops. Pepsine, and other drugs also, are often ruined by the manipulations; therefore the dissatisfaction we so often have to complain of, and the cause of our resorting to less elegant and simpler articles. The so-called "chicken pepsine" is simply the tough mucous lining of the common fowl's gizzard or triturating stomach, which is removed in preparing that organ for the table, dried and pulverized. If used fresh we need only to give from 25 to 30 grains (adult) of the powder, but if kept it is liable to spoil; therefore I suspend it in alcohol when not used in fresh powder, and am satisfied that it has done better than any preparation that I have bought, and I can refer, I believe, to several physicians who use it more than any other.

